# Computer-assisted subcapital correction osteotomy in slipped capital femoral epiphysis using individualized drill templates

**DOI:** 10.1186/s41205-021-00108-6

**Published:** 2021-07-06

**Authors:** Sima Zakani, Christopher Chapman, Adam Saule, Anthony Cooper, Kishore Mulpuri, David R. Wilson

**Affiliations:** 1 Department of Pediatrics, Faculty of Medicine, University of British Columbia, Vancouver, BC Canada; 2 Department of Orthopaedic Surgery, SUNY Downstate Medical Center, Brooklyn, NY USA; 3 Department of Mechanical Engineering, Dalhousie University, Halifax, NS Canada; 4 Department of Orthopaedic Surgery, BC Children’s Hospital, Vancouver, BC Canada; 5 Department of Orthopaedics, University of British Columbia, Vancouver, BC Canada

## Abstract

**Background:**

Subcapital osteotomy by means of surgical hip dislocation is a treatment approach offered for moderate-to-severe cases of Slipped Capital Femoral Epiphysis (SCFE). This procedure is demanding, highly dependent on the surgeon’s experience, and requires considerable radiation exposure for monitoring and securing the spatial alignment of the femoral head. We propose the use of individualized drill guides as an accurate method for placing K-wires during subcapital correction osteotomies in SCFE patients.

**Methods:**

Five CT scans of the hip joint from otherwise healthy patients with moderate-to-severe SCFE were selected (ages 11–14). Three dimensional models of each patient’s femur were reconstructed by manual segmentation and physically replicated using additive manufacturing techniques. Five orthopaedic surgeons virtually identified the optimal entry point and direction of the two threaded wires for each case. 3D printed drill guides were designed specific to each surgical plan, with one side shaped to fit the patient’s bone and the other side containing holes to guide the surgical drill. Each surgeon performed three guided (using the drill guides) and three conventional (freehand) simulated procedures on each case. Each femur model was laser scanned and digitally matched to the preoperative model for evaluation of entry points and wire angulations. We compared wire entry point, wire angulation, procedure time and number of x-rays between guided and freehand simulated surgeries.

**Results:**

The guided group (1.4 ± 0.9 mm; 2.5° ± 1.4°) was significantly more accurate than the freehand group (5.8 ± 3.2 mm; 5.3° ± 4.4°) for wire entry location and angulation (*p* < 0.001). Guided surgeries required significantly less drilling time and intraoperative x-rays (90.5 ± 42.2 s, 3 ± 1 scans) compared to the conventional surgeries (246.8 ± 122.1 s, 14 ± 5 scans) (*p* < 0.001).

**Conclusions:**

We conclude that CT-based preoperative planning and intraoperative navigation using individualized drill guides allow for improved accuracy of wires, reduced operative time and less radiation exposure in simulated hips.

**Clinical relevance:**

This preliminary study shows promising results, suggesting potential direct benefits to SCFE patients by necessitating less time under anesthesia and less intra-operative radiation exposure to patients, and increasing surgical accuracy.

## Background

Slipped capital femoral epiphysis (SCFE) is a common adolescent hip disorder, affecting 1 in 10,000 to 20,000 children [1–5]. The pathogenesis of SCFE is believed to be multifactorial; obesity [6–8] and abnormal morphology [4] at the hip joint have been shown to play a contributing role.

Of the several treatment options for SCFE [9], it is known that surgical techniques permitting anatomic reduction and stabilization of the slipped epiphysis with no damage to the blood supply have the best long-term outcomes [1, 10]. Subcapital osteotomy by means of surgical hip dislocation is one such treatment option offered for moderate-to-severe cases of SCFE [10] that provides the ability to monitor the vascular flow in the femoral head [11]. Intraoperative fluoroscopy is used in different stages of the surgery, particularly for monitoring the spatial alignment of the femoral head and drilling the threaded wires. While clinical outcomes of these surgeries are generally good [12–14], this technique is not widely performed due to its demanding nature, high dependency on the surgeon’s experience and the considerable intra-operative radiation exposure to the young patient.

Computer-assisted methods such as opto-electronic navigation can help overcome the limitations of subcapital osteotomy by improving screw placement accuracy [15] and significantly reducing intraoperative x-ray exposure. However, they require extra equipment, namely the opto-electronic tracking system [16], and increase operating time [15–17] as necessitated by intraoperative registration between the patient and the preoperative image data. Individualized instrument guides can overcome these drawbacks. Used in other technically difficult orthopaedic procedures, [16, 18–24] an instrument guide contains built-in holes or cutting lines specific to the patient’s anatomy and the surgeon’s pre-operative plan, thus eliminating the need for tracking equipment and intraoperative registration. The guides are 3D-printed pre-operatively using bio-compatible materials and sterilized for use in the operating room.

The purpose of this study was to determine the radiographic outcomes for using patient-specific drill templates in a series of simulated subcapital osteotomy surgeries.

## Materials and methods

### Materials

The feasibility and accuracy of the proposed method was tested in a laboratory setting. Five pre-operative CT scans of the hip joint from otherwise healthy patients with moderate-to-severe SCFE were used. CT scans were of one male and four females aged 11–14 years; three left hips and two right hips were included. Each of the five hip joints were CT scanned at a slice thickness of 0.625 mm. Three dimensional (3D) models of each patient’s proximal femur were reconstructed by importing the CT dataset using the commercially available Mimics software (Materialise, Leuven, Belgium) and manual segmentation of the bony anatomy. The segmented model for each part of the femur anatomy (head and neck/shaft) was saved as tessellated surface (STL) format for further use.

### Study design

**Fig. 1:**
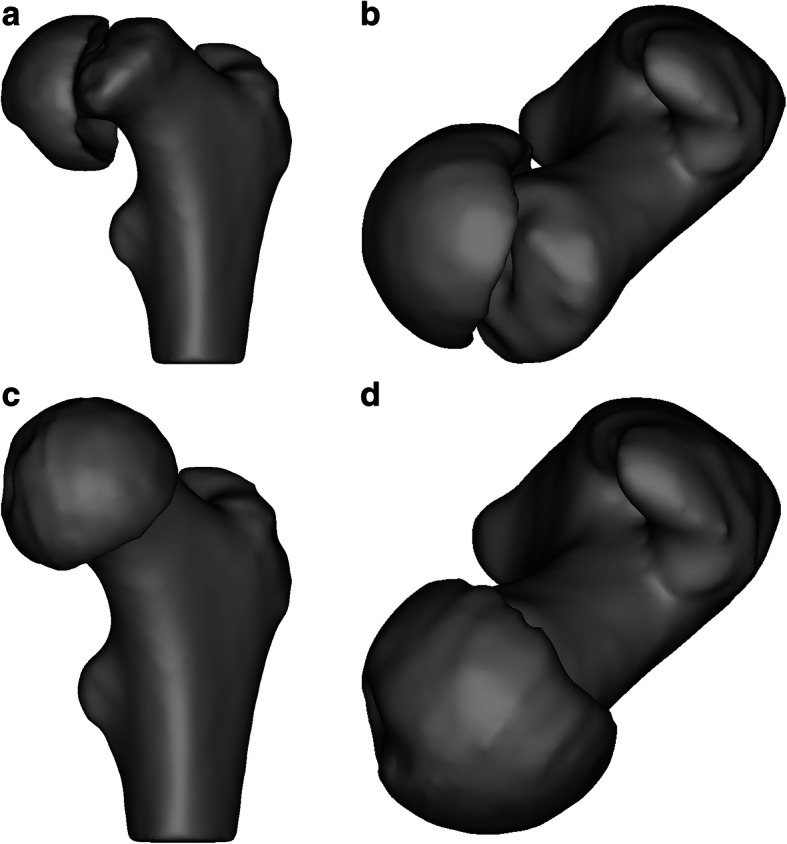
Spatial alignment of the femoral head with respect to femoral neck in its anatomic state of one of the hips included in the study (**a**. anterior, **b**. superior) and after reduction (**c**. anterior, **d**. superior) following general guidelines of subcapital osteotomy in SCFE

Five orthopaedic surgeons participated in this study: two experienced pediatric orthopaedic surgeons, two pediatric orthopaedic clinical fellows, and one third-year orthopaedic resident. One surgeon (co-author CC) defined the optimal position of the femoral head (head reduction) for each of the five 3D reconstructed femurs following general guidelines of the subcapital correction osteotomy in SCFE [10], as shown in Fig. 1. Each surgeon performed thirty simulated surgeries on the patient-specific plastic femur models; fifteen conventional (freehand) and fifteen guided, using a custom drilling guide (see specifications later).

The surgical phase in this study is defined as steps following femoral head reduction:

#### Step 1

Drilling a K-wire through the fovea such that it exits the lateral cortex slightly below the trochanter.

#### Step 2

Drilling two K-wires, uniformly distributed in the head, in antegrade technique to secure the position of the head.

**Fig. 2:**
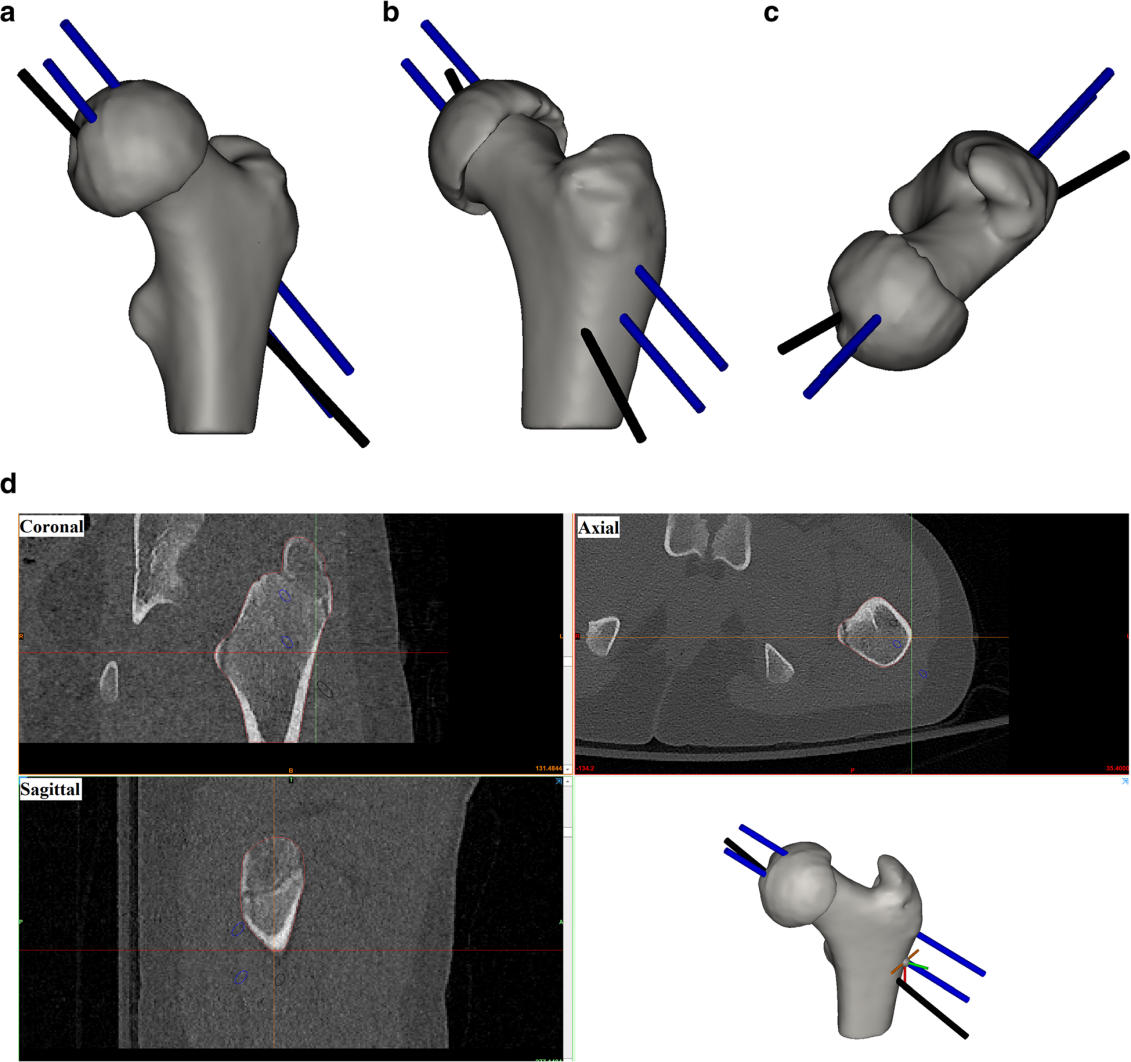
Virtual pre-operative planning in a representative case (**a**. anterior, **b**. lateral, **c**. superior). Each surgeon identified the optimal position for three k-wires in the three anatomical planes, one through the fovea and two uniformly distributed in the head using Mimics (**d**. Mimics interface)

In this procedure the foveal wire is considered to be a temporary measure to secure the reduced head in place prior to drilling the two non-foveal wires. As such, accurate placement of the foveal wire beyond the fact that it should exit the lateral cortex slightly below the trochanter is not a key aspect of this surgery. The surgeons planned the placement of the two non-foveal wires for each of the reduced femurs using computer-aided design (CAD) models of 3-mm K-wires [10] imported into Mimics. The planning process included identifying the optimal placement for each wire and positioning it accordingly (Fig. 2).

**Fig. 3:**
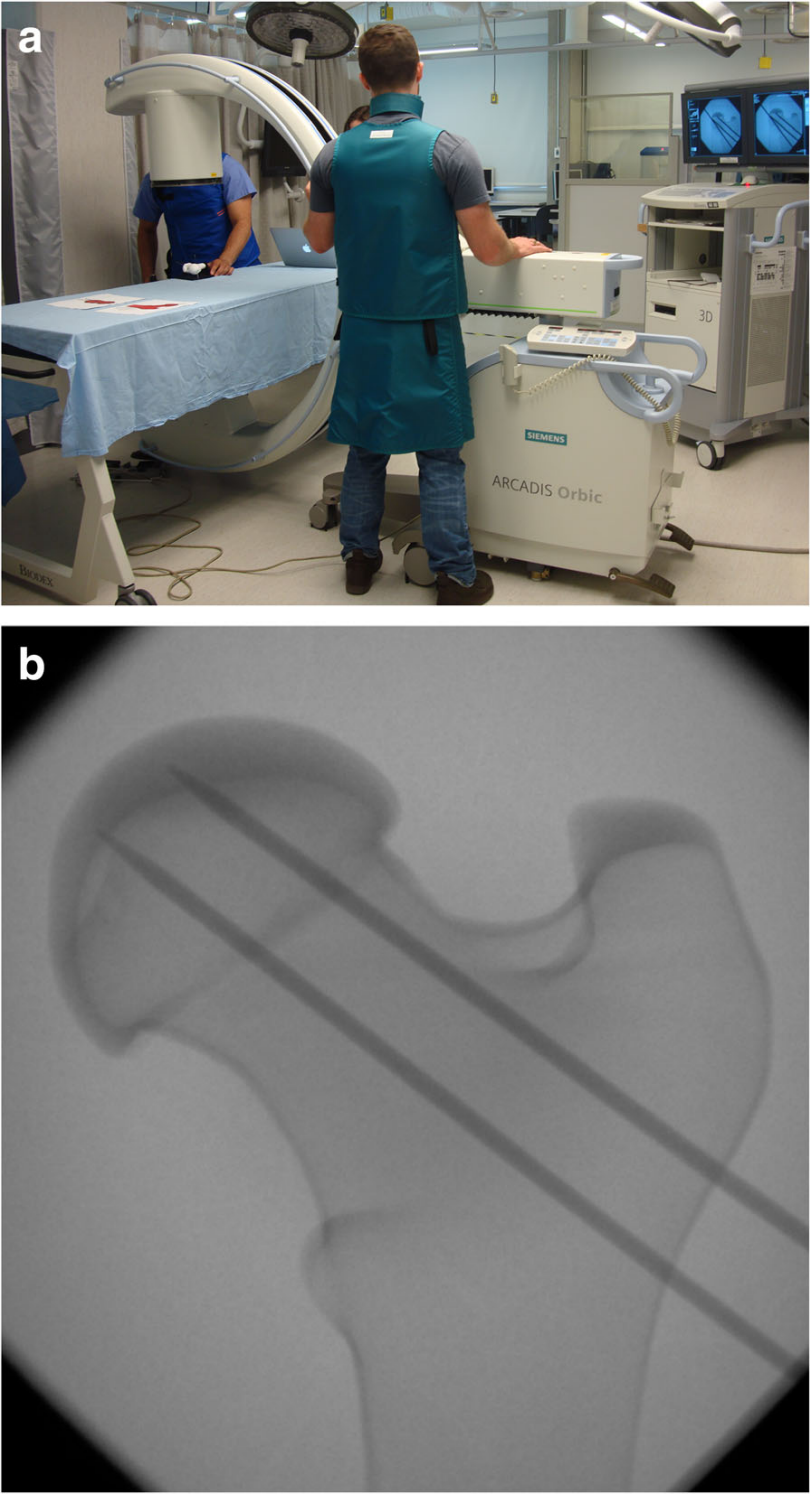
Data collection set-up; **a**. Surgeons used a c-arm for intra-operative validation, as needed; **b**. The contrast in radiodensity of casted bone models and the wires allowed for visualization of the bony contour and wire trajectory during the simulated surgeries

For the freehand surgeries, surgeons were asked to replicate the pre-operative plan, with access to antero-posterior and lateral views of their pre-operative plan but were blind to the optimal length of wires as is typical in a conventional surgery. For the guided surgeries, surgeons used a patient-specific drill guide specifically designed for their pre-operative plan for placement of the two non-foveal wires and were given the optimal length for each wire placement that had been determined based off their pre-operative plan. To remove the possibility of self-optimization and learning on the task, the sequence of surgeries for each surgeon was assigned randomly. Surgeons used a c-arm fluoroscope (Arcadis Orbic 3D, Siemens, Munich, Germany) for intra-operative validation, as needed (Fig. 3). Each surgery was timed, and the number of intra-operative x-rays was recorded. After surgery, each model was laser-scanned (ES 360, Afinia 3D, Minnesota, United States) and reconstructed in 3D to compare the final wire position with the preoperative plan. Two separate scans of each model were acquired to capture all facets of the model (~ 3 min each). The scans were then stitched together in the scanner’s proprietary software and exported as an STL file for further processing.

### Drilling platform design and subject-specific model creation

**Fig. 4:**
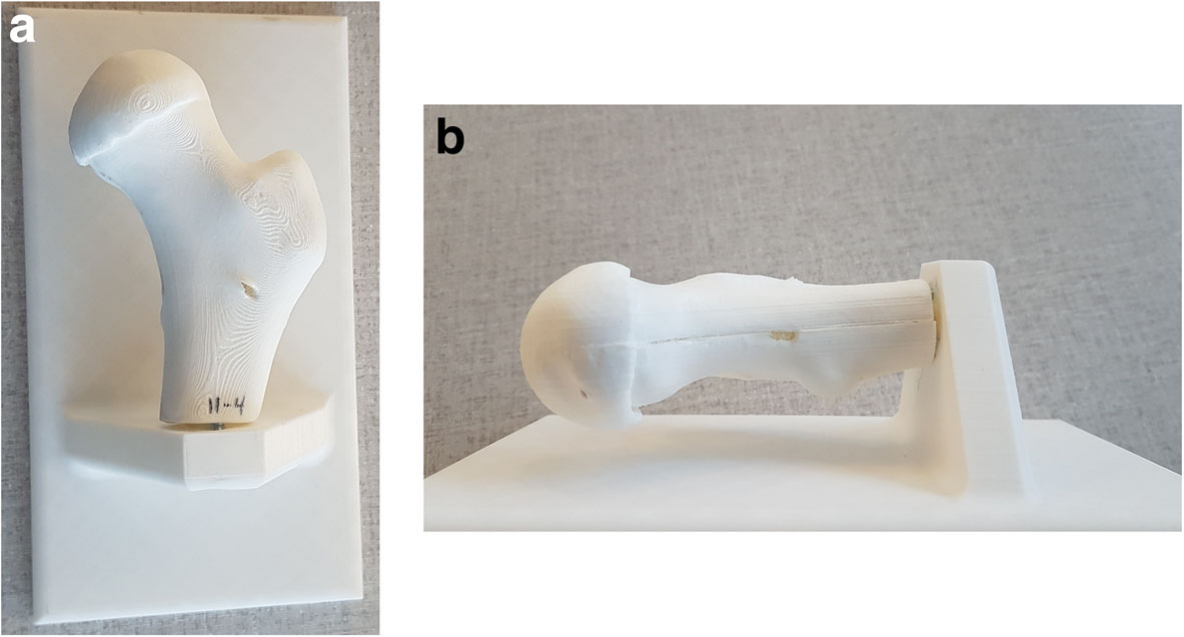
Drilling platform for simulated surgeries (**a**. view from top, **b**. view from right). The platform consisted of a 3D printed fixed support and an interchangeable bone unit. The two parts are attached using screws

**Fig. 5:**
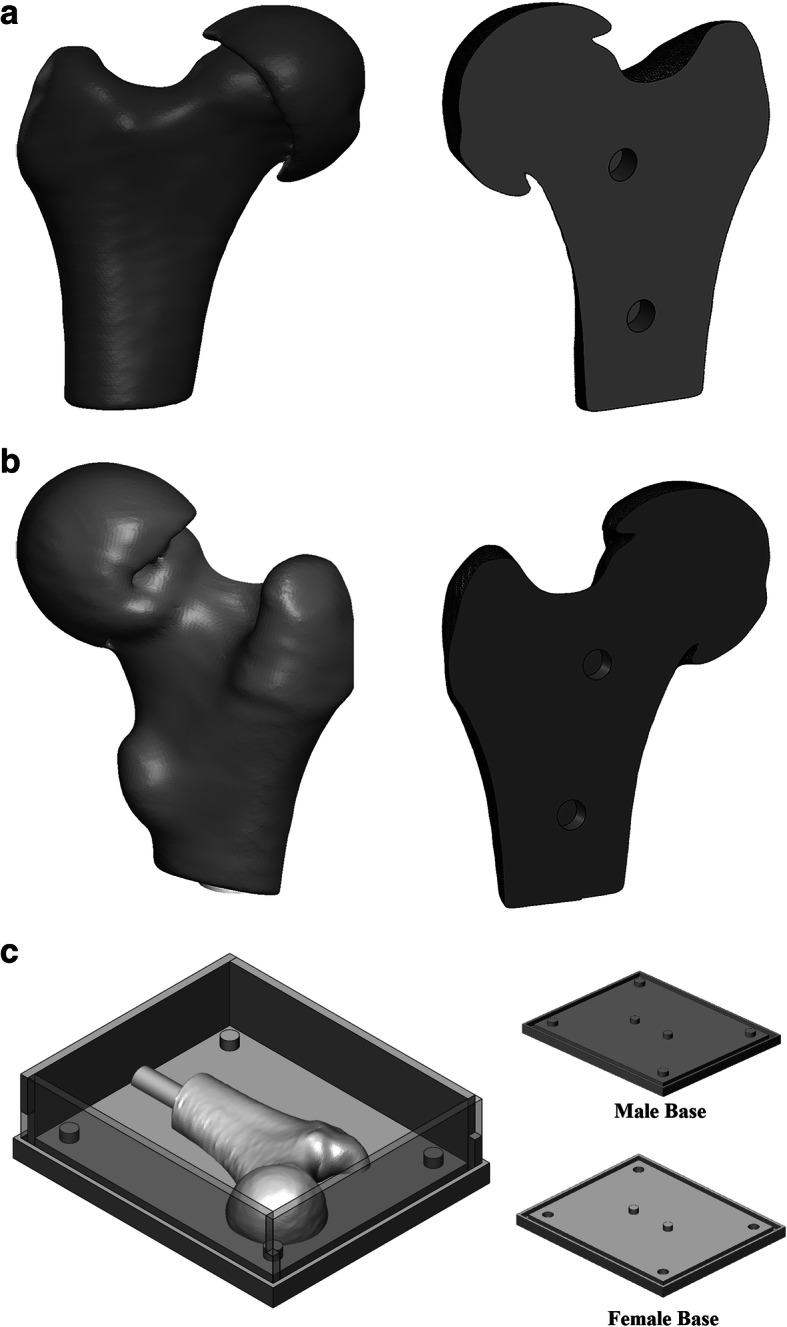
Digital models of the femur and mold box; Each bone was digitally divided into two halves (**a**. anterior, **b**. posterior) to be later molded into urethane rubber (note the holes for alignment pins in the split surface that ensures secure registration to the base of mold box); **c**. two separate mold boxes were designed to account for male and female halves of the final rubber mold (note the difference in alignment pins)

**Fig. 6:**
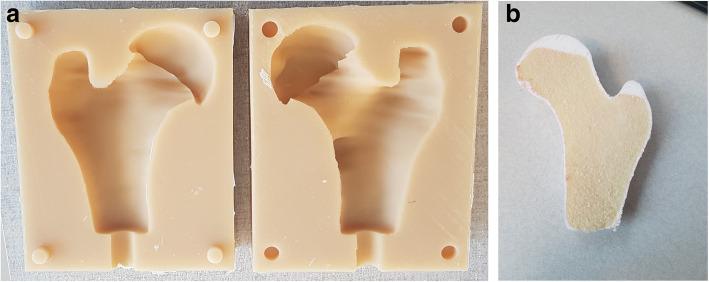
Bone models were created using urethane rubber mold and two different urethane casting resins; **a**. The two halves of the urethane rubber mold; **b**. example bone model cut in half to demonstrate the semi-rigid shell and porous foam mimicking real bone structure

Surgeries were performed on a drilling platform consisting of an interchangeable patient-specific proximal femur and a fixed support fixture securing the distal end and elevating the femur model during the simulated surgeries, Fig. 4. The support fixture was printed in polylactic acid (PLA) using a 3D printer (Ultimaker 2, Ultimaker B.V., Utrecht, Netherlands). The 3D model of each patient’s femur was virtually split into anterior and posterior sections and printed in PLA with the mating surface placed on the print platform to avoid support attachments on anatomical features, Fig. 5a. The molds were created using a custom assembly, consisting of interchangeable male and female bases with alignment pins embedded, and four interlocking walls, Fig. 5b. Each half femur was secured to the base of mold assembly using matching alignment pins and was molded into urethane rubber to create a negative print, Fig. 6a. Each mold was used to cast thirty copies of the corresponding femur. The cast model was made using two different urethane casting resins. To simulate the cortical shell, 40 mL of a semi-rigid casting resin (Smooth Cast™ 65D, Smooth-On Inc., Pennsylvania, United States) was poured into the mold and rotated for five minutes to ensure even coating of the mold surface. The resin volume was estimated based on the surface area of each 3D femur and a wall thickness of 2 mm. The shell was allowed to harden for an extra five minutes before pouring 35 mL of an expanding urethane foam (Foam-iT!™ 5, Smooth-On Inc., Pennsylvania, United States) simulating cancellous bone, Fig. 6b. To avoid uncontrolled changes to the anatomical features no manual surface post-processing was performed on either the 3D printed femur models or respective casted bone models.

### Creation of patient-specific drill guides

For each simulated guided surgery, the 3D reduced femur and the K-wire placement and pathway planned by each surgeon were imported into computer aided design (CAD) software (SolidWorks, Dassault Systèmes, Vélizy-Villacoublay, France). Surgeons were asked to select a registration surface on the respective femur considering the surgical approach, soft tissue layers, and registration stability [18]. Subcapital correction osteotomy in SCFE through surgical hip dislocation provides access to the anterior aspect of the proximal femur [10] exposing the antero-superior surface of the femoral neck with limited access to the antero-lateral surface of the femoral shaft below the greater trochanter. This in turn provided a registration surface that could be cleaned during the surgery without permanent damage as well as adequate anatomical features to allow for stable positioning of the drill guide.

**Fig. 7:**
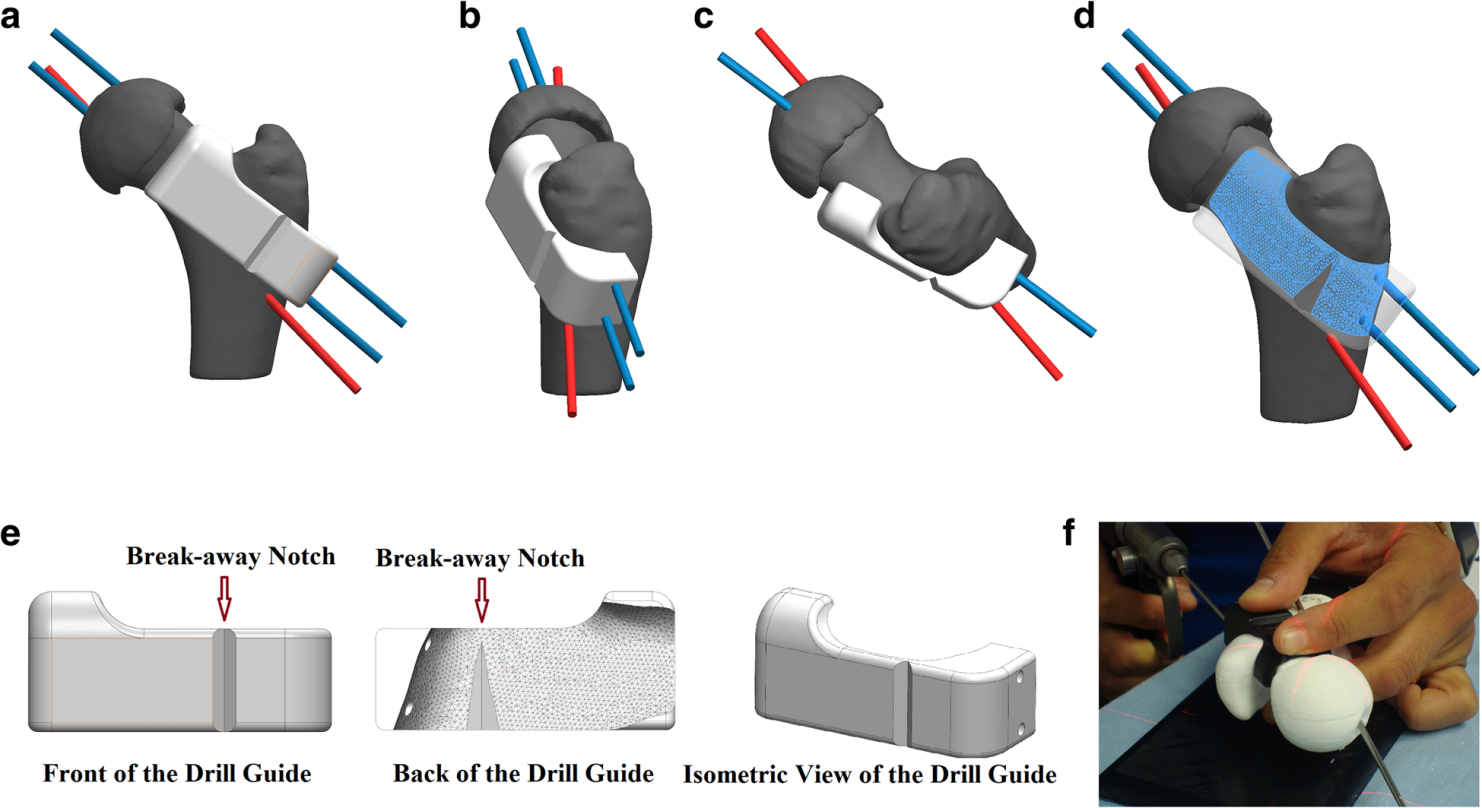
Registration surface for a patient-specific instrument is guided by surgical exposure of proximal femur following standard of care; **a**. anterior registration surface is selected to ensure clearance from head-neck junction, fit patient’s femoral-shaft anatomy (starting below the greater trochanter and ending above the foveal wire’s exit point); **b**. lateral registration surface accommodates wire distribution; **c**. superior registration surface relies on surgical access to femoral neck’s mid-line; **d**. overall registration surface; **e**. final instrument design with break-away notch to ensure easy removal; **f**. placement and use of a representative drill guides in a simulated surgery

To ensure registration surface stability the drill guide was designed to wrap around the sub-trochanteric femoral shaft (ending at the femoral mid-line) and the antero-superior side of the femoral neck (ending at the neck mid-line; Fig. 7a and b). Using Solidworks, the registration surface as well as CAD K-wire paths were subtracted from a virtual drill guide (0.2 mm tolerance for all surfaces), resulting in a patient-specific drill guide mimicking the pre-operative plan. A notch was created on both anterior and posterior sides of the guide to ensure smooth removal after drilling (Fig. 7c and d). Each patient-specific guide was saved in a tessellated surface format (STL format) and printed, using the same printer, in polylactic acid (PLA), a biocompatible and biodegradable polymer with minor inflammatory reactions reported to-date [25, 26]. Surface fit between each 3D printed guide and its respective 3D printed femur model was used as a qualitative measure of quality control.

### Post-operative evaluation

Once the simulated surgery was complete, each bone model (with K-wires drilled) was laser-scanned using a tabletop 3D scanner (Afinia, Microboards Technology LLC, Minnesota, US). The scanned bone models were registered to the matching CT reconstructed models in Meshlab [27] in a two-step process: (i) a paired-point registration using easily identified anatomical landmarks, such as the greater trochanter, lesser trochanter, and fovea followed by (ii) a surface registration including all the points in both models. The resulting transformation matrix was then imported into MATLAB (MathWorks, Massachusetts, US) and a shape-matching analysis [28] was performed to compare the laser-scanned 3D reconstructions of casted bone models with the original 3D femur models. The simulated drilling characteristics (entry point and angulation) and resulting accuracy were then compared to the preoperative planning model. For each wire, the entry or exit points were defined as the mid-point of three points along the perimeter of the wire as it enters from the lateral aspect or exits from the femoral head. The Euclidean distance between wire entry points in the pre-operative plan and post-operative results was calculated as:

$$ d=\sqrt{{\left({x}_{post- entry}-{x}_{pre- entry}\right)}^2+{\left({y}_{post- entry}-{y}_{pre- entry}\right)}^2+{\left({z}_{post- entry}-{z}_{pre- entry}\right)}^2} $$

Wire direction (for both the pre-operative plan and the post-operative results) was identified as the unit-vector of the line connecting the entry point to the exit point of each wire:

$$ \hat{u}=\frac{\left({x}_{exit},{y}_{exit},{z}_{exit}\right)-\left({x}_{entry},{y}_{entry},{z}_{entry}\right)\ }{\sqrt{{\left({x}_{exit}-{x}_{entry}\right)}^2+{\left({y}_{exit}-{y}_{entry}\right)}^2+{\left({z}_{exit}-{z}_{entry}\right)}^2}} $$

The angulation error was defined as the angle between the two unit-vectors defining the wire angulation in the pre-operative plan and post-operative results and calculated as:

$$ \theta =\arctan \left(\frac{{\hat{u}}_{pre}\times {\hat{u}}_{post}}{{\hat{u}}_{pre}.{\hat{u}}_{post}}\right) $$

### Statistical analysis

The characteristics of 300 K-wires (2 wires × 150 bone models) were compared with their matching pre-operative plan. Accuracy was defined as the closeness of the drilling characteristics to the pre-operative plan.

The non-parametric rank sum test, with *p* < 0.05 as the significance criterion, was used to compare accuracy of wire entry points and angulation as well as intra-operative parameters such as surgical duration and number of x-rays between the freehand and guided surgeries.

## Results

**Fig. 8:**
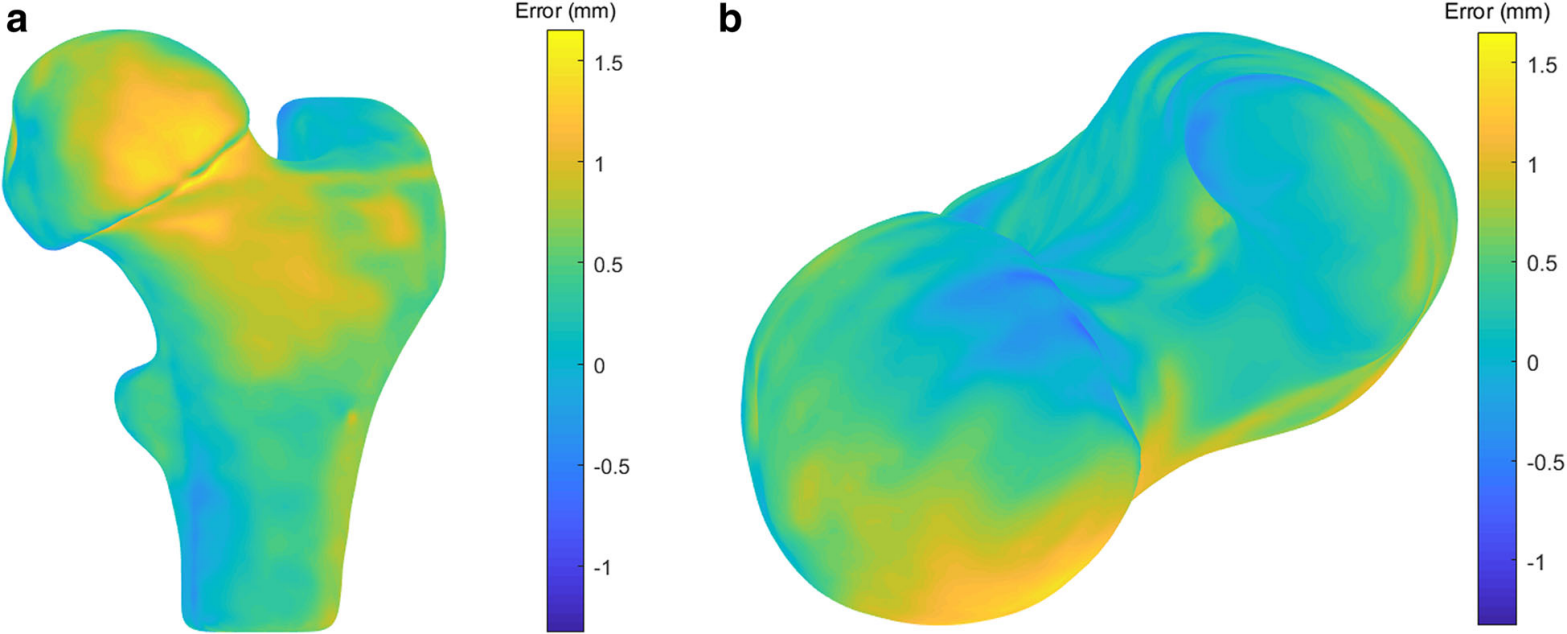
Topographic map of a representative casted bone used to verify shape consistency and accuracy in **a**. anterior and **b**. superior views

The shape-matching analysis [28] comparing the laser-scanned 3D reconstructions of casted bone models with the original 3D femur models revealed an average root mean square error (RMSE) and standard deviation of 0.5 ± 0.2 mm, Fig. 8.

**Fig. 9:**
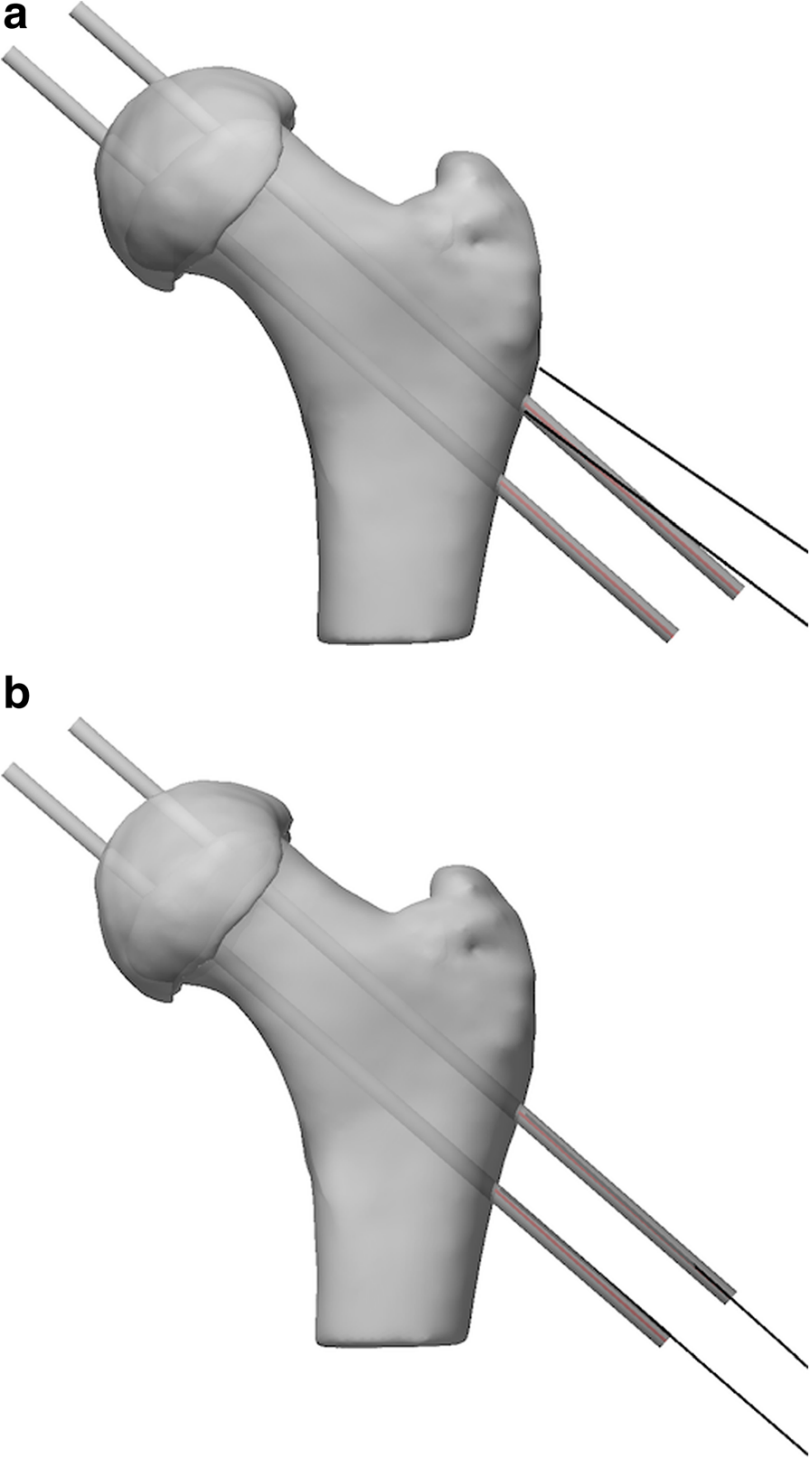
Visual representation of drilling path error in anterior plane in a representative freehand simulated surgery (**a**) vs a guided simulated surgery (**b**). The black lines represent the trajectory of wires according to plan. The red lines represent the results from the simulated surgeries

The wire entry point for the guided group was significantly more accurate (1.4 ± 0.9 mm) than the freehand group (5.8 ± 3.2 mm) (*p* < 0.001). Wire angulation was significantly more accurate for the guided group (2.5° ± 1.4°) compared to the freehand group (6.3° ± 4.4°)(*p* < 0.001). Figure 9 shows the drilling outcome from one surgeon on a representative bone, in freehand (9.a) and guided (9.b) scenarios. The red and black lines represent the pre-operative plan and the drilled path taken by the same surgeon, respectively. Details of drilling parameters for each wire can be found in Table 1. Post-hoc sample size analysis was conducted to determine the minimum number of patients required to achieve a power of 0.9 with a significance of 0.05 considering the null value of wire entry point accuracy and wire angulation for the freehand group (5.8 ± 3.2 mm and 6.3° ± 4.4°, respectively) and the mean of reported values for the guided group (1.4 mm and 2.5°). Calculations led to a sample size of 15 which is far surpassed with the current sample size of 75 in each group.

**Table 1:** Drilling parameters comparing freehand and guided surgery K-wire placement compared to the pre-operative plan for both wires (superior and inferior) and pooled by group

Drilling Parameters	Wire	Freehand Mean ± STD	Guided Mean ± STD	*p* value
Wire Entry Point (mm)	Superior	5.7 ± 3.2	1.2 ± 0.8	**< 0.001**
	Inferior	5.9 ± 3.3	1.6 ± 1.0	**< 0.001**
	Pooled	5.8 ± 3.3	1.4 ± 0.9	**< 0.001**
Wire Angulation (degrees)	Superior	6.6 ± 4.3	2.4 ± 1.5	**< 0.001**
	Inferior	6.0 ± 4.5	2.5 ± 1.3	**< 0.001**
	Pooled	6.3 ± 4.4	2.5 ± 1.4	**< 0.001**

Statistically significant *p* values (*p* < 0.05) are in bold. Pooled data include values reported for both views. Compared to freehand surgeries, guided surgeries were statistically significantly more accurate in both drilling parameters

Table 2 summarizes intra-operative characteristics such as duration of surgery and number of x-rays used for drilling validation. Guided surgeries required significantly less drilling time and fewer intraoperative x-rays (1.5 ± 0.7 min; 3 ± 1 scans) compared to the freehand group (4.1 ± 2.0 min; 14 ± 5 scans) (*p* < 0.001). Of the 75 freehand simulated surgeries, 32 required at least a second attempt at drilling one of the wires, while only five of the 75 guided surgeries required a second attempt.

**Table 2:** Intra-operative characteristics comparing freehand and guided surgery K-wire placement compared to the pre-operative plan

Intra-operative Characteristics	Direction	Freehand Mean ± STD	Guided Mean ± STD	*p* value
Duration (minutes)		4.1 ± 2.0	1.5 ± 0.7	**< 0.001**
Number of X-rays	Antero-posterior	10 ± 3	2 ± 1	**< 0.001**
	Medio-lateral	4 ± 3	1 ± 1	**< 0.001**
	Pooled	14 ± 5	3 ± 1	**< 0.001**

Statistically significant *p* values (*p* < 0.05) are in bold. Pooled data include values reported for both views. Compared to freehand surgeries, guided surgeries were significantly faster and required less numbers of intra-operative x-rays

## Discussion

We developed patient-specific drill guides for placement of the two non-foveal wires in subcapital osteotomy by means of hip dislocation. These guides improved the accuracy of wire entry point positioning and angulation during these surgeries and reduced surgery time and the number of intraoperative radiographs needed. These improvements facilitate better bone purchase and wire distribution within the neck, potentially creating a more secure femoral head fixation.

The wire entry point and angulation were of particular interest as these wires dictate the position and orientation of final cannulated screws or threaded wires securing the position of the femoral head. These screws receive the majority of the joint load, limit further slipping of the femoral head and maintain the femoral anatomical position. Although the wire entry zones on the antero-lateral side of the proximal femur are readily visible by surgeons, there are few anatomical landmarks to guide the drill path, making this procedure a good candidate to be improved by patient-specific drill guides.

A further advantage of the patient-specific drill guides is that they reduced intra-operative fluoroscopic imaging by 80%. Although the use of fluoroscopic imaging is the most harmful to patients, it also puts the clinical staff at a greater risk. Attempts at reducing radiation risk are often targeted towards better training for the clinical staff, software solutions for imaging algorithms, or hardware solutions with lower radiation dose [29]. These solutions are often costly and have variable outcomes. Patient-specific drill guides reduce radiation risk by allowing surgeons to perform K-wire placement with less need for intra-operative imaging.

The procedure time is another indicator of efficiency provided by the subject-specific drill guides: the guided method averaged 2.6 min (60%) faster than the freehand method. Although the drill guides did not guide the foveal wire placement, they acted as visual cues for foveal wire placement where they were used. In the absence of such visual help in freehand simulated surgeries, the number of re-drills due to intersecting wire trajectories were higher. The decreased number of errant drill passes may explain the reduced operating time and may potentially have the advantage of maintaining the integrity of the femoral neck bone stock.

One limitation of this study is that it was performed in a controlled laboratory environment with casted plastic bone models based on medical images from a small number of patients, representing the ideal surgical exposure without the need for cleaning soft tissues surrounding the femoral neck and greater trochanter. Manual assessment of range of motion is another essential part of traditional subcapital correction osteotomy in SCFE through surgical hip dislocation which also increases operative time. Therefore, our findings may not be representative of freehand or guided surgeries in an operating room environment. Further study is needed to assess the impact on clinical outcomes on larger cohorts of patients. Nevertheless, our findings are in line with the literature suggesting that guided surgeries can enhance operative efficiency and accuracy [16, 18, 24, 30, 31]. With the use of casted bone models of similar size and weight, the reduced number of images acquired was assumed to correlate to reduced overall radiation per case. However, in a real surgical environment with patients of varying weight and size, technique factors are a preferable metric for measuring radiation exposure. Future study is also needed to compare the radiation exposure due to intra-operative fluoroscopy with pre-operative CT acquisition. This method is recommended for cases with a pre-operative CT scan of the hip readily available. Future work optimizing the guide thickness for appropriate constriction of the wires, printing with bio-compatible and sterilisable material, and studying clinical outcomes in a randomized control trial would be valuable.

To conclude, CT-based preoperative planning and intraoperative use of patient-specific drill guides improve the accuracy of wire placement in subcapital correction osteotomies in simulated moderate-to-severe SCFE hips. Improved accuracy with less operative time and fewer intraoperative images conveys a potential direct benefit to SCFE patients by necessitating less time under anesthesia and less intraoperative radiation exposure to patients.

## Data Availability

The datasets used and/or analysed during the current study are available from the corresponding author on request.
